# Acetylsalicylic acid versus clopidogrel in cardiovascular prevention: Does the classic still outperform the contemporary?

**DOI:** 10.17179/excli2026-9251

**Published:** 2026-02-12

**Authors:** Ian Jhemes Oliveira Sousa, Kerolayne de Melo Nogueira

**Affiliations:** 1 Northeast Biotechnology Network (RENORBIO), Federal University of Piauí (UFPI), Teresina, Piauí, Brazil; 2 Postgraduate Program in Physiology and Pharmacology, Federal University of Ceará (UFC), Fortaleza, Ceará, Brazil

## ⁯

The discussion regarding the optimization of antiplatelet therapy for the secondary prevention of major adverse cardiovascular events has been revisited, with the replacement of acetylsalicylic acid (ASA) by P2Y12 receptor inhibitor monotherapy, such as clopidogrel, increasingly promoted as the new recommended strategy (Capodanno et al., 2018[2]; Watanabe et al., 2024[11]). Although recent literature, driven by individual patient data meta-analyses and randomized clinical trials, suggests a potential superior effectiveness of clopidogrel monotherapy in reducing ischemic events (Valgimigli et al., 2025[10]), it is crucial for the clinical body and the scientific community to adopt a cautious stance before abandoning the nearly century-old therapeutic pillar that is ASA. In this letter, we emphasize that the duality between the classic strategy and new trends should not be resolved by uncritical adherence to results that, while statistically significant, lack a broader context and an in-depth analysis of methodological limitations and clinical implications.

ASA, an irreversible inhibitor of cyclooxygenase-1 (COX-1), has its efficacy in secondary prevention established by decades of use and robust meta-analyses demonstrating a consistent reduction in the risk of serious vascular events (Antithrombotic Trialists' Collaboration, 2002[1]; Reilly and FitzGerald, 1988[8]). The longevity and universality of its use, combined with its cost-effectiveness, confer upon it a status that cannot be disregarded. The primary criticism of ASA lies in its safety profile, specifically the increased risk of gastrointestinal (GI) bleeding with prolonged use (Sostres et al., 2013[9]). However, this limitation is often presented out of context.

The risk of GI bleeding is dose-dependent, and in current clinical practice, the doses of ASA used for cardiovascular prevention are the lowest possible and frequently combined with gastric protection strategies, such as enteric-release formulations or even proton pump inhibitors (PPIs) in high-risk patients (Lanas et al., 2007[6]). Simply replacing ASA with clopidogrel under the premise of a more favorable GI safety profile ignores the fact that clopidogrel, also being an antiplatelet agent, carries a bleeding risk, and the difference in major bleeding risk between the two agents in monotherapy has not been shown to be statistically significant in large studies (Valgimigli et al., 2025[10]).

One of the most cited and recent pieces of evidence in favor of clopidogrel monotherapy comes from the meta-analysis by Valgimigli et al. (2025[10]), which reported a significant reduction in major adverse cardiovascular and cerebrovascular events (MACCE) with clopidogrel compared to ASA in patients with established coronary artery disease (CAD). Although the hazard ratio (HR) of 0.86 for MACCE is notable, it is crucial to examine the composition of the included studies. Most patients in these trials had undergone percutaneous coronary intervention (PCI), and in many cases, clopidogrel monotherapy was initiated after a period of dual antiplatelet therapy (DAPT) (Hahn et al., 2019[4]). This scenario differs substantially from pure secondary prevention, where ASA has been the standard maintenance treatment. Direct extrapolation of these results to all patients in secondary prevention, regardless of PCI history, is a risky simplification. Furthermore, the lack of statistical difference in mortality and major bleeding between the groups suggests that the marginal ischemic benefit of clopidogrel may not translate into a superior net clinical benefit, especially when considering the robustness and cost-effectiveness of ASA.

The duality is further complicated when considering antiplatelet resistance. ASA resistance, defined as the failure to achieve adequate platelet inhibition in vivo, is a documented phenomenon that can lead to unfavorable ischemic outcomes (Ibrahim et al., 2013[5]). However, clopidogrel is not exempt from this problem. Response variability to clopidogrel, driven by genetic polymorphisms of cytochrome P450 2C19 (CYP2C19), is a well-established clinical challenge, resulting in a significant portion of patients being slow or intermediate metabolizers who cannot efficiently convert the prodrug into its active metabolite (Mega et al., 2009[7]). A study evaluating resistance in patients with Acute Coronary Syndrome (ACS) demonstrated that clopidogrel resistance may be more prevalent than ASA resistance (Ibrahim et al., 2013[5]). Replacing ASA with clopidogrel, therefore, may simply exchange one resistance problem for another, potentially more prevalent and pharmacogenetically complex to manage.

The trend toward de-escalation of DAPT, exemplified by the SMART-CHOICE trial (Hahn et al., 2019[4]), which demonstrated the non-inferiority of P2Y12 inhibitor monotherapy (mainly clopidogrel) after only 3 months of DAPT versus prolonged DAPT, is an important advance in reducing bleeding risk. However, this study does not directly compare clopidogrel monotherapy versus ASA monotherapy in the long term. It only validates the safety of early ASA discontinuation, but the choice of maintenance agent (clopidogrel) is based on the premise that the P2Y12 inhibitor is the more potent component and thus more suitable for maintenance. This premise, while reasonable in the post-PCI context where stent thrombosis is a primary concern, should not be generalized to the entire secondary prevention population.

The SMART-CHOICE 3 study (Choi et al., 2025[3]), which directly compared clopidogrel monotherapy versus ASA monotherapy in high-risk patients after PCI, is often cited as definitive evidence of clopidogrel's superiority. However, it is fundamental to note that the observed ischemic benefit was modest, and again, the absence of difference in mortality and major bleeding should temper irresponsible enthusiasm. Treatment decisions should always weigh individual ischemic risk against hemorrhagic risk. For patients with lower ischemic risk or those for whom treatment cost is a barrier, ASA remains a perfectly valid and clinically effective therapeutic option, with a safety profile that can be effectively managed with prophylactic measures.

In summary, the literature advocating for the replacement of ASA with clopidogrel monotherapy in secondary prevention, while growing, must be interpreted with a critical eye. The historical robustness and cost-effectiveness of ASA, the lack of difference in safety and mortality outcomes compared to clopidogrel, and the complexity of clopidogrel resistance suggest that the classic strategy still holds a central role. Therapeutic duality does not imply the obsolescence of ASA but rather the need for more refined individualization of treatment. Clopidogrel monotherapy should be reserved for subgroups of patients who demonstrate a clear and significant ischemic benefit, such as those with high post-PCI ischemic risk, and not as a universal replacement for ASA. Clinical prudence requires that the classic, proven, and accessible strategy be maintained as the foundation of antiplatelet treatment, while new strategies are carefully integrated to optimize care in specific scenarios.

## Declaration

### Conflict of interest

The authors declare that there are no commercial, financial, or personal relationships that could be constructed as a potential conflict of interest regarding this article.

### Artificial Intelligence (AI) - assisted technology

The authors declare that no artificial intelligence tools were used in the writing or revision of this manuscript.

## References

[1] Antithrombotic Trialists' Collaboration (2002). Collaborative meta-analysis of randomised trials of antiplatelet therapy for prevention of death, myocardial infarction, and stroke in high risk patients. BMJ.

[2] Capodanno D, Mehran R, Valgimigli M, Baber U, Windecker S, Vranckx P (2018). Aspirin-free strategies in cardiovascular disease and cardioembolic stroke prevention. Nat Rev Cardiol.

[3] Choi KH, Park YH, Lee JY, Jeong JO, Kim CJ, Yun KH (2025). Efficacy and safety of clopidogrel versus aspirin monotherapy in patients at high risk of subsequent cardiovascular event after percutaneous coronary intervention (SMART-CHOICE 3): a randomised, open-label, multicentre trial. Lancet.

[4] Hahn JY, Song YB, Oh JH, Chun WJ, Park YH, Jang WJ (2019). Effect of P2Y12 Inhibitor Monotherapy vs Dual Antiplatelet Therapy on Cardiovascular Events in Patients Undergoing Percutaneous Coronary Intervention: The SMART-CHOICE Randomized Clinical Trial. JAMA.

[5] Ibrahim O, Oteh M, A Syukur A, Che Hassan HH, S Fadilah W, Rahman M (2013). Evaluation of Aspirin and Clopidogrel resistance in patients with Acute Coronary Syndrome by using Adenosine Diposphate Test and Aspirin Test. Pak J Med Sci.

[6] Lanas A, García-Rodríguez LA, Arroyo MT, Gomollón F, Feu F, González-Pérez A (2006). Risk of upper gastrointestinal ulcer bleeding associated with selective cyclo-oxygenase-2 inhibitors, traditional non-aspirin non-steroidal anti-inflammatory drugs, aspirin and combinations. Gut.

[7] Mega JL, Close SL, Wiviott SD, Shen L, Hockett RD, Brandt JT (2009). Cytochrome p-450 polymorphisms and response to clopidogrel. N Engl J Med.

[8] Reilly IA, FitzGerald GA (1988). Aspirin in cardiovascular disease. Drugs.

[9] Sostres C, Gargallo CJ, Lanas A (2013). Nonsteroidal anti-inflammatory drugs and upper and lower gastrointestinal mucosal damage. Arthritis Res Ther.

[10] Valgimigli M, Choi KH, Giacoppo D, Gragnano F, Kimura T, Watanabe H (2025). Clopidogrel versus aspirin for secondary prevention of coronary artery disease: a systematic review and individual patient data meta-analysis. Lancet.

[11] Watanabe H, Morimoto T, Natsuaki M, Yamamoto K, Obayashi Y, Nishikawa R (2024). Clopidogrel vs Aspirin Monotherapy Beyond 1 Year After Percutaneous Coronary Intervention. J Am Coll Cardiol.

